# Nanoemulsions Enhance *in vitro* Transpapillary Diffusion of Model Fluorescent Dye Nile Red

**DOI:** 10.1038/s41598-019-48144-x

**Published:** 2019-08-14

**Authors:** Samantha L. Kurtz, Louise B. Lawson

**Affiliations:** 1 Department of Microbiology and Immunology, Tulane University School of Medicine, New Orleans, LA 70112 USA; 2 Bioinnovation Ph.D. Program, Tulane University School of Science and Engineering, New Orleans, LA 70118 USA

**Keywords:** Microscopy, Drug delivery, Breast cancer

## Abstract

While the feasibility of transpapillary drug delivery has previously been established, localized transport via the mammary ducts may be improved with tailored drug delivery formulations. The objective of this study was to investigate the impact of nanoemulsion encapsulation on transpapillary delivery *in vitro*. Nanoemulsion formulations composed of isopropyl myristate and Tween 80 encapsulating a fluorescent dye were applied topically on porcine nipples using a Franz diffusion cell. A combination of dye extraction and fluorescence image analysis was used to quantify the total amount of dye retained within the nipple and to characterize the penetration routes. After diffusion for 6 hours, the amount of dye deposited in the nipple was proportional to the formulation’s water concentration. The 90% water formulation deposited significantly more dye via both the stratum corneum and mammary ducts, while the 80% and 70% water formulations moderately increased ductal penetration, but minimally altered stratum corneum penetration as compared to the control solution. Similar trends were found after diffusion for 48 hours; however, the overall impact was diminished, likely due to the nanoemulsion’s topical instability. This study indicates that drug delivery vehicles, nanoemulsions specifically, enhance delivery of encapsulated molecules via the stratum corneum and mammary ducts in a formulation-dependent basis.

## Introduction

The incidence of non-invasive breast cancer, namely ductal carcinoma *in situ*, has increased 5-fold in the past 3.5 decades^1^, concomitant with the widespread use of mammography, and now accounts for 20% of all breast cancer diagnoses^2^. An even higher incidence of women are diagnosed with non-obligate precursor lesions such as atypical hyperplasia^3–5^. Despite an increased incidence of localized disease and a better understanding of its biology, the available treatment approaches, including a combination of surgery, radiation, chemotherapy or hormone therapy, could better be tailored for this patient population. The long- and short-term psychological and physical side effects associated with these treatments are important considerations for the 60–70% of women whose disease will not progress to invasive breast cancer^5,6^. Therefore, the estimated 160,000 women in the United States diagnosed annually with pre-malignant or non-invasive breast cancer^2,5^ may benefit from a localized treatment approach that avoids first-pass metabolism and thus systemic effects, while maximizing drug exposure within the breast.

An optimal approach would directly expose the epithelial cells that line the mammary ductal network to a chemopreventive agent. These epithelial cells represent only 8% of the total cellular makeup of the mammary gland^7^, but are the cell population that most commonly malignantly transforms, accounting for 95% of breast cancer cases^8^. We and others have demonstrated that diffusion through and retention within the mammary canals varies depending on a molecule’s and drug carrier’s physicochemical properties^9–11^. Specifically, lipophilic small molecules preferentially diffuse through the conduits, capitalizing on the ductal orifices as an entry point and transport pathway. Furthermore, drug carriers with diameters of at least 5 nm injected intraductally remain concentrated within the mammary ducts^9,12^, thereby limiting exposure within the surrounding breast stroma and distant extraneous organs. Enhanced targeting of the ductal orifices and passive diffusion through the canals may therefore be accomplished through design of tailored drug delivery vehicles.

Microemulsion and nanoemulsion delivery platforms are the most commonly used system for topical skin technologies^13^ and have more recently become a promising approach for cancer prevention and treatment^14^. Their two-phase composition of immiscible liquids, oil and water, and a surfactant stabilizer yields droplets that are typically <1 μm in size, isotropic, and thermodynamically (microemulsions) or kinetically (nanoemulsions) stable^15,16^. The selection and proportion of each component drives spontaneous formation of one of three different microemulsion types: water-in-oil, oil-in-water, or bicontinuous^17^. For transfollicular applications, microemulsions with greater water content preferentially target the hair follicles more so than vehicles with lower water content or a control solution^18^. The advantages of micro- and nanoemulsions for topical/skin delivery has been reviewed by Sutradhar and Amin^19^ and Nastiti *et al*.^20^, both of which highlight the advantages of emulsion-based delivery and the influence of emulsion formulation on transport properties. The ability of emulsion systems to enhance penetration into a skin orifice suggests their potential advantage for transpapillary delivery as well.

A previous study of microemulsion diffusion through the nipple indicated varied tissue retention and permeation depending on the microemulsion formulation^21^. A 5% water formulation significantly decreased drug retention within the nipple without impacting permeation as compared to an ethanolic solution, while a formulation with 35% water significantly increased both drug retention and permeation. However, the specific routes of penetration were not addressed^21^. Therefore, the targeting capabilities within the ductal channels of emulsion-based formulations remain unknown. Our own previous investigation of transpapillary delivery, in which we used qualitative and quantitative assessments to analyze permeation at both a micro and macro scale, indicates that permeation occurs via two distinct pathways, transepidermal and transductal^9^. Herein, we sought to use similar techniques to elucidate the role of emulsion-encapsulation on transport via the mammary papillae.

Based on the ability of microemulsions to enhance transport via hair follicles and the mammary papillae on a formulation-dependent basis, we hypothesized that an emulsion-based formulation with high water content would improve the efficiency of ductal transport. The objective of the current study was to evaluate the impact of nanoemulsion encapsulation on transpapillary drug delivery, and in particular, transport into the mammary ducts using three nanoemulsion formulations of varying water concentrations. Each formulation encapsulated nile red (NR), a model fluorescent lipophilic dye, for ease of quantification and visualization of the penetration pathways.

## Results and Discussion

### Physicochemical properties of nanoemulsions

To determine the relative water, oil, and surfactant concentrations yielding a nanoemulsion using isopropyl myristate (IPM) and Tween 80 (T80) as the oil phase and surfactant, respectively, we systematically varied the concentrations of each component. A limited nanoemulsion domain was found in the area of high water and surfactant content, which is consistent with previous findings^22,23^. Three formulations with water percentages ranging from 70–90% along the 1:9 oil:surfactant dilution line were chosen for characterization and *in vitro* transpapillary diffusion (Supplementary Fig. S1). Although no attempt was made to verify the microstructure, based on the high proportion of water in each formulation and the aqueous solubility of T80 (HLB = 15^22^), the nanoemulsions were assumed to be of oil-in-water type^17^.

Nanoemulsion droplets loaded with the lipophilic small molecule NR (MW = 318.37 Da; logP = 5) were characterized for size, dispersity, and zeta potential. Each formulation had similar average diameters ranging from 9.57 to 12.30 nm (Table 1), which was confirmed using cryogenic transmission electron microscopy (cryoTEM) (Supplementary Fig. S2). The polydispersity index (PDI) increased as the water content decreased, transitioning from a more monodisperse droplet population to a broader size distribution. Meanwhile, the zeta potential of the nanoemulsions ranged from −0.16 to −0.01 mV, indicating their neutral charge^24^. The droplets in each formulation were considered nanoemulsions, rather than microemulsions, as the size distributions were more heterogeneous (PDI > 0.1^25,26^) and instability was observed following long-term storage at room temperature (Supplementary Fig. S3)^27^, which are both factors that distinguish nanoemulsions from microemulsions.

**Table 1:** Physicochemical properties of nanoemulsion formulations.

Oil (%)	Surfactant (%)	Water (%)	Diameter (nm)	PDI	Zeta Potential (mV)
1	9	90	11.91 ± 0.73	0.33 ± 0.07	−0.13 ± 0.15
2	18	80	12.30 ± 2.32	0.51 ± 0.13	−0.01 ± 0.32
3	27	70	9.57 ± 2.15	0.65 ± 0.18	−0.16 ± 0.32

Data are represented as mean ± SEM (n = 3).

### Total penetration of NR into the nipple

**Figure 1:**
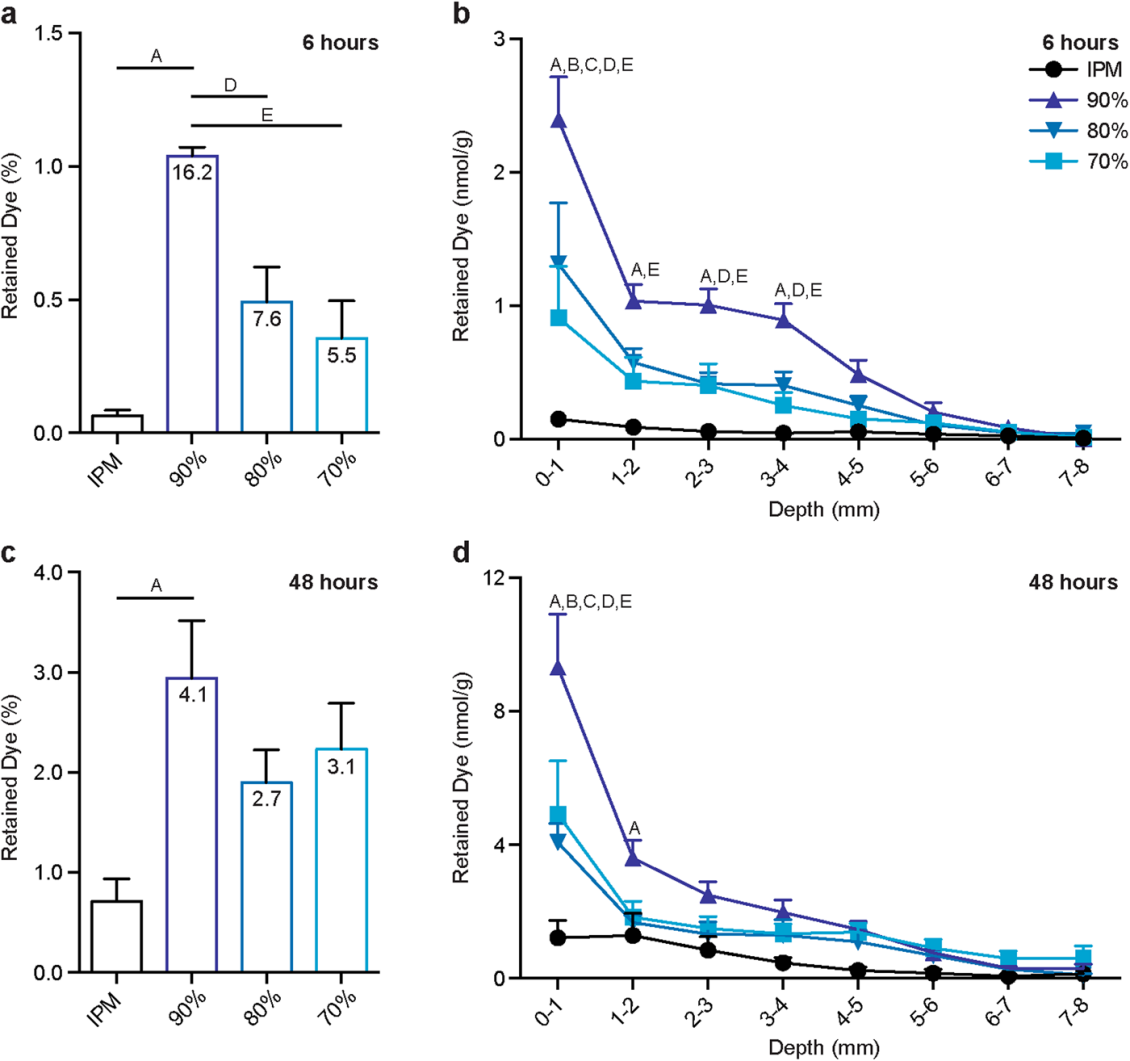
Penetration of NR into porcine nipples *in vitro* after topical application for 6 (**a**,**b**) or 48 (**c**,**d**) hours. The numbers within the bars (**a**,**c**) represent the fold-change as compared to NR penetration from the IPM solution (black bar). Data is expressed as % of total applied dye (**a**,**c**) or delineated by depth (**b**,**d**). Each data point represents mean + SEM (n = 6). Statistical analysis was performed with one-way ANOVA (Retained Dye %) or two-way ANOVA (Retained Dye nmol/g) with Tukey’s multiple comparisons post-test. p ≤ 0.05 was considered significant and is denoted using the following indicators: A- IPM vs 90%, B- IPM vs 80%, C- IPM vs 70%, D- 90% vs 80%, E- 90% vs 70%, F- 80% vs 70%.

To understand the impact of nanoemulsion encapsulation on dye penetration into the nipple, NR was extracted from tissue following diffusion of nanoemulsions or the control solution. After diffusion for 6 hours, only 0.06% of NR from the control IPM solution was deposited within the nipple (Fig. 1). The nanoemulsion formulations, however, increased penetration of NR 5.5 to 16.2-fold, depending on the nanoemulsion composition, to 0.36–1.04% of the total amount applied. Similar trends were identified after diffusion for 48 hours, with the nanoemulsion formulations depositing more NR into the tissue compared to the IPM solution, however the differences were less pronounced. Approximately 0.70% of NR from the IPM solution was retained within the nipple compared to 1.90–2.94% of NR from the nanoemulsion formulations, corresponding to 2.7 to 4.1-fold increases.

When assessing dye retention as a function of tissue depth, the main differences between treatments were found in the uppermost tissue sections (Fig. 1b,d), where the highest amount of dye was deposited. After 6 hours of diffusion, the 90% water nanoemulsion formulation deposited significantly more dye from 1–4 mm in nipple depth compared to the IPM solution and the other nanoemulsion formulations (Fig. 1b). After 48 hours of diffusion, the same trend was found, however, only in the upper-most 1 mm of tissue (Fig. 1d). The 70% and 80% water nanoemulsions formulations deposited significantly more NR only in the uppermost 1 mm of tissue as compared to the control solution, but did so at both the 6- and 48-hour time point. As tissue depth increased, the amount of dye deposited in the tissue decreased for each treatment, which is consistent with previous reports^9,28^.

Among the nanoemulsion formulations, a direct relationship was found between the percentage of water in the formulation and the amount of deposited NR following 6 hours of diffusion (Fig. 1a). The amount of NR retained within the tissue for each formulation followed the order 90% > 80% > 70%. However, after 48 hours, the trend slightly shifted to 90% > 70% > 80%, albeit no significant differences were found among the nanoemulsion formulations at this time point (Fig. 1b). Even within the relatively small range of water concentrations in our formulations, the water content substantially influenced penetration into both the nipple and skin. While the influence of water content on nanoemulsion-based transpapillary delivery has not been previously assessed, our findings are consistent with studies that focused on the impact of water content on microemulsion transport. A transpapillary diffusion study of varying microemulsion formulations reported significantly more retention of α-santalol (logP = 4.5) within the nipple from a 35% water content formulation than a 5% water content formulation^21^. Similarly, skin penetration and permeation of numerous lipophilic compounds from oil-in-water microemulsions with high aqueous percentages is more pronounced than from microemulsions with lower water content^18,29–31^, thereby increasing drug retention in both the stratum corneum and viable skin layers up to 3.7-fold^30^. The ability of microemulsions to increase skin hydration^29^ resulting in ultrastructural changes in the tissue that make stratum corneum transport more favorable^32^ coupled with copious amounts of the penetration enhancer water^29,33^ likely contributes to the improved deposition of drugs within tissue.

### Microscopic evaluation of NR penetration

**Figure 2:**
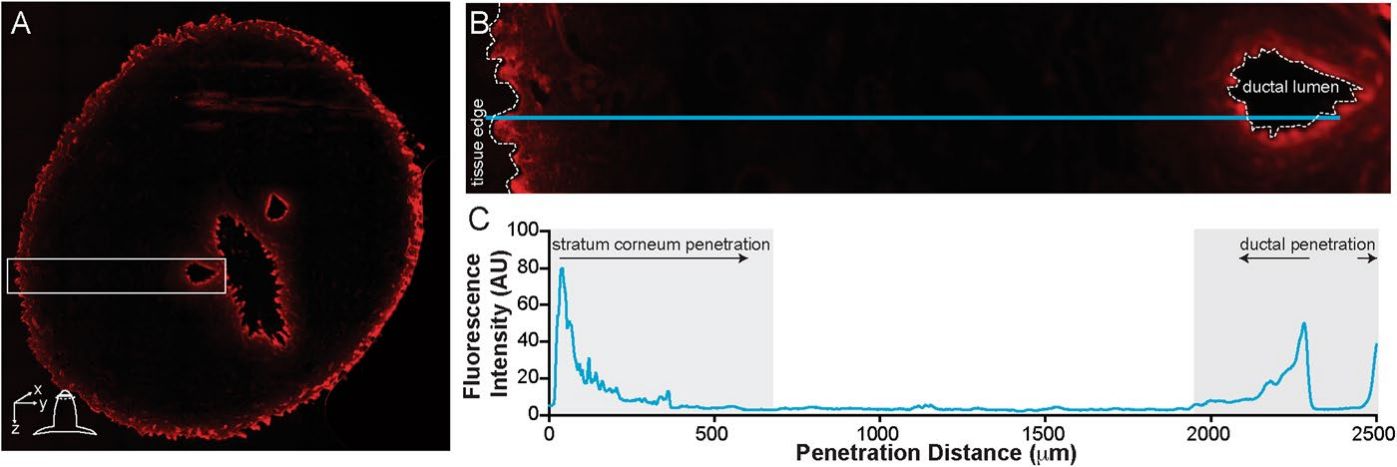
Generation of fluorescence intensity profiles using ImageJ plot profile. A fluorescence montage of a porcine nipple cross-section (**A**) was used for all image analysis. The line tool (**B**, blue line) was drawn from either the stratum corneum edge or the ductal edge towards the inner connective tissue. The plot profile function was then used to generate the fluorescence pixel intensity as a function of distance along the line. (**C**) For each penetration route, 5–8 intensity profiles were averaged per tissue section or duct.

Previous studies illustrate varied utilization of two penetration routes into the nipple, transepidermal and transductal, depending upon the physicochemical properties of the diffusing small molecule or drug vehicle^9,10,28^. To microscopically evaluate the penetration differences between the nanoemulsion formulations and assess distinct penetration routes, image analysis was performed on fluorescence micrographs of nipple cross-sections. The plot profile tool in ImageJ was used to generate fluorescence intensity as a function of distance from either the stratum corneum edge (stratum corneum penetration) or the mammary duct edge (ductal penetration) into the connective tissue (Fig. 2). From this plot, the penetration distance and the peak fluorescence intensity was quantified. This analysis was performed at consecutive nipple depths. A schematic representation of transpapillary diffusion as a function of nipple depth and penetration distance is available in Kurtz *et al*.^28^. Representative fluorescence micrographs used in image analysis are provided in Supplementary Figs S4 and S5.

#### Penetration of NR via the nipple ducts

**Figure 3:**
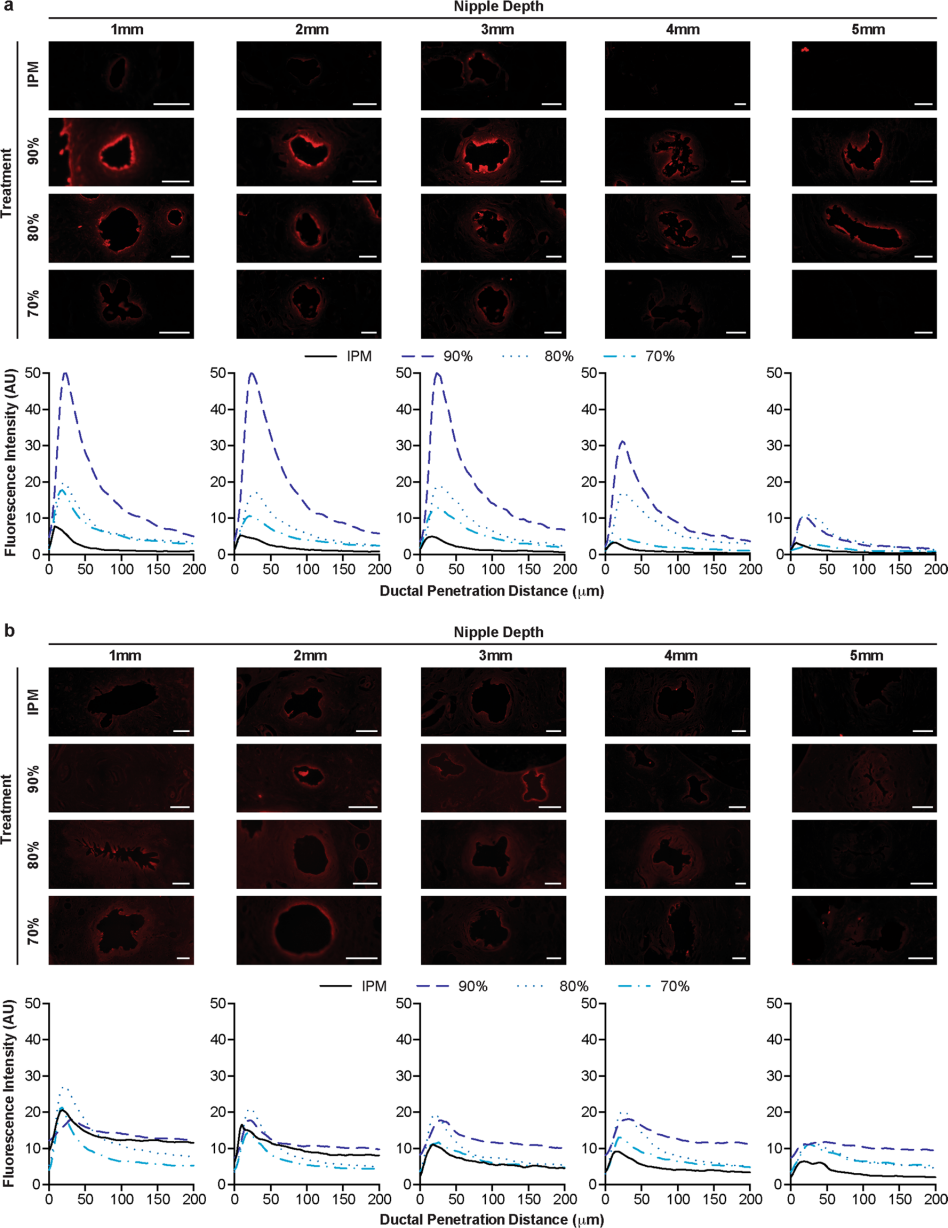
Representative fluorescence micrographs of mammary ducts and corresponding fluorescence intensity profiles following diffusion of nanoemulsions or control IPM solution for 6 (**a**) or 48 (**b**) hours. White scale bars represent 250 μm. Graphs are mean of at least quintuplicate measures of n = 6 nipples.

After topical application for 6 hours, fluorescence was detected from the IPM solution approximately 50 μm into the connective tissue from the ductal edge at every nipple depth (Fig. 3a). In contrast, fluorescence from the nanoemulsion formulations extended well beyond this distance, penetrating upwards of 200 μm at nipple depths 1–4 mm or 100 μm at 5 mm, suggesting that the nanoemulsions improved NR diffusion laterally into the connective tissue from the mammary ducts after only 6 hours. However, after diffusion for 48 hours, there were minimal differences in the penetration distance between the topical treatments (Fig. 3b). Every treatment, the nanoemulsions and control solution, had at least a low level of fluorescence exhibited at 200 μm into the connective tissue. Furthermore, the fluorescence intensity within the connective tissue was sustained from 75–200 μm, suggesting that a low level of saturation was reached.

**Figure 4:**
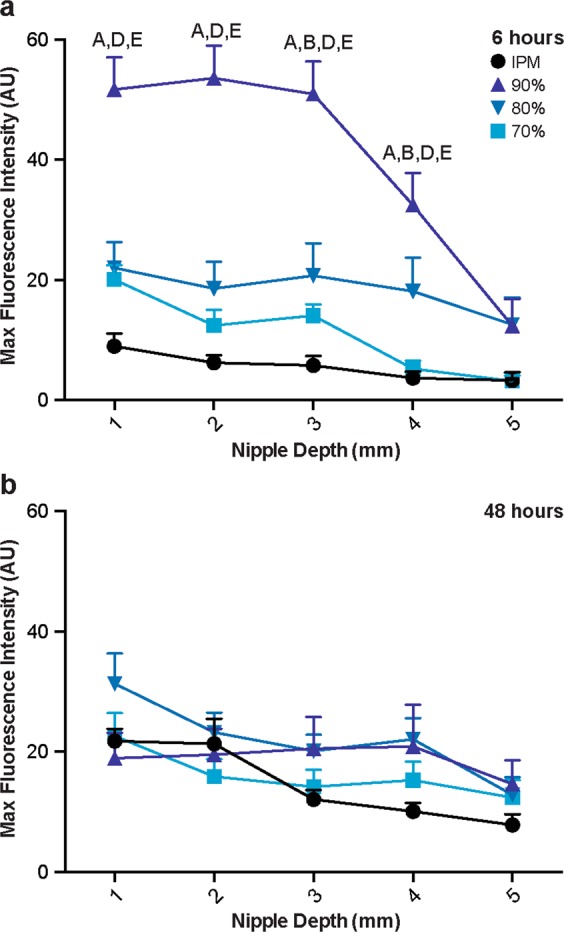
Peak fluorescence intensity of NR at the ductal edge of porcine nipples treated with nanoemulsions or an IPM solution for 6 (**a**) or 48 (**b**) hours. Data is represented as mean + SEM (n = 8–12). Statistical analysis was performed with two-way ANOVA with Tukey’s multiple comparisons post-test. p ≤ 0.05 was considered significant and is denoted using the following indicators: A- IPM vs 90%, B- IPM vs 80%, C- IPM vs 70%, D- 90% vs 80%, E- 90% vs 70%, F- 80% vs 70%.

The transition from stark differences in the penetration distance between treatments after diffusion for 6 hours to minimal differences after 48 hours was consistent for peak fluorescence intensities, as well. After diffusion for 6 hours, the peak fluorescence intensity (occurring at the ductal edge around 25 μm) was higher for the nanoemulsion formulations, particularly for the 90% water formulation, than the IPM solution at nearly every tissue depth (Figs 3a and 4a). Within the top 3 mm, all nanoemulsion formulations had higher peak fluorescence intensities than the IPM solution, indicating that higher concentrations of NR were deposited within the mammary channels for extended depths. At 4 mm in depth, the peak fluorescence intensity from the formulation with the lowest water content (70%) decreased to a level that was similar to the IPM solution. By 5 mm in nipple depth, the peak fluorescence intensities from the 80% and 90% water emulsions had also decreased as compared to the upper tissue regions, but were still slightly higher than the IPM solution. However, after diffusion for 48 hours, no significant differences were detected between any of the treatments, suggesting that each treatment had similar ductal NR concentration profiles (Fig. 3b). Previous studies indicate that microemulsions enhance transfollicular penetration after diffusion for 24 hours as compared to a control solution^18^. While these results are consistent with our 6 hour time point, the targeting capability of nano and microemulsions are likely dependent upon formulation and stability. However, both their and our results suggest these drug delivery vehicles are adept at quickly targeting orifices on the surface of both the nipple and skin.

Furthermore, between 6 and 48 hours of diffusion, the peak fluorescence intensities at the ductal edge either notably decreased (90% water formulation), remained relatively unchanged (80% and 70% water formulations) or increased (IPM solution) (Fig. 4). The penetration distance, however, increased over time for every treatment suggesting progression towards equilibrium, as previously reported^9^. Image analysis kinetics of an ethanolic NR solution or sulforhodamine B solution in phosphate buffered saline (PBS) showed increased penetration distances over time with varied ductal edge fluorescence intensities, yet both solutions had a substantial reduction in the plot profile slope, indicative of the concentration gradient, between 30 minutes and 8 hours when diffusing via the ductal penetration pathway.

#### Penetration of NR via the nipple stratum corneum

**Figure 5:**
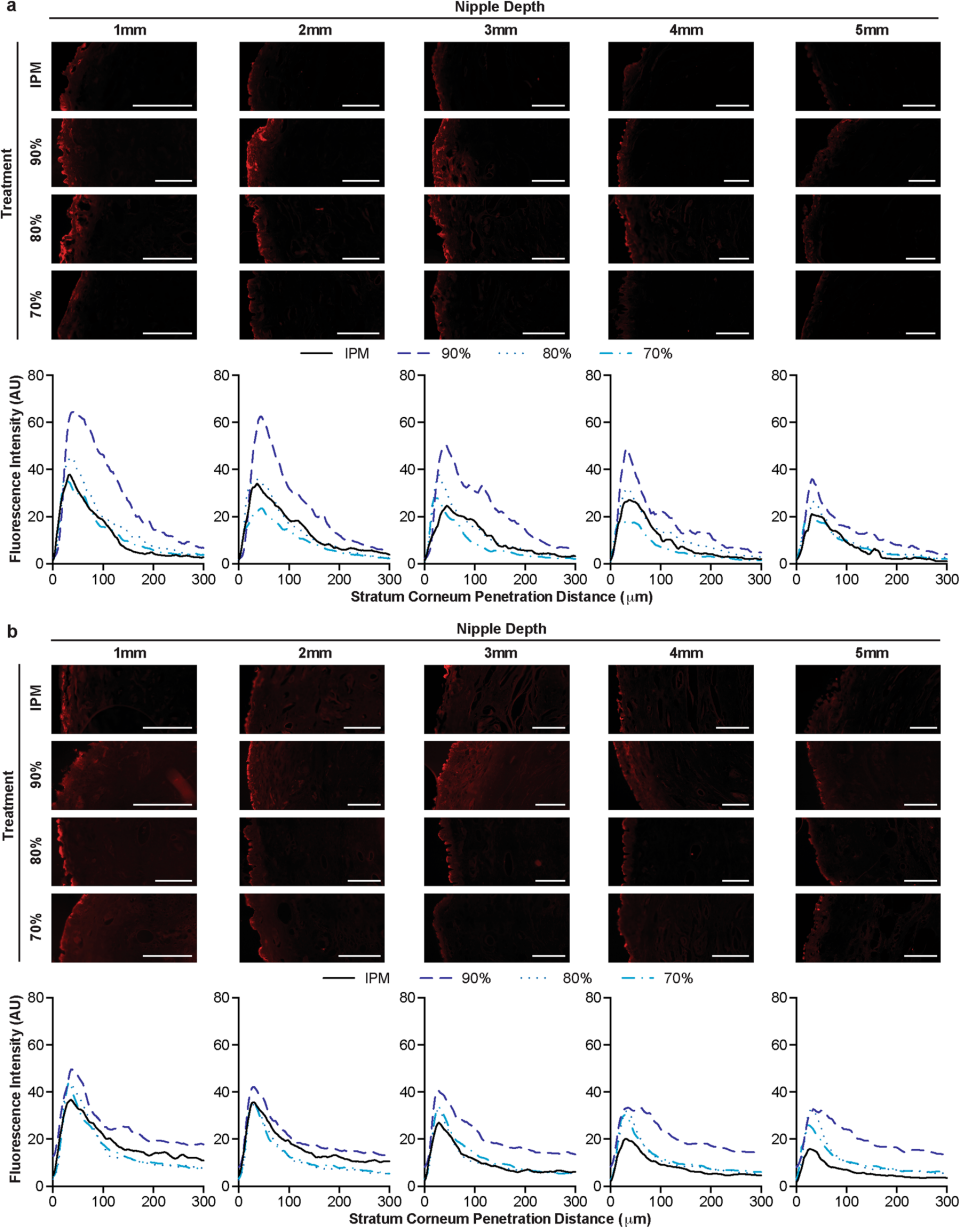
Representative fluorescence micrographs of the stratum corneum and corresponding fluorescence intensity profiles following diffusion of nanoemulsions or control IPM solution for 6 (**a**) or 48 (**b**) hours. White scale bars represent 500 μm. Graphs are mean of at least quintuplicate measures of n = 6 nipples.

**Figure 6:**
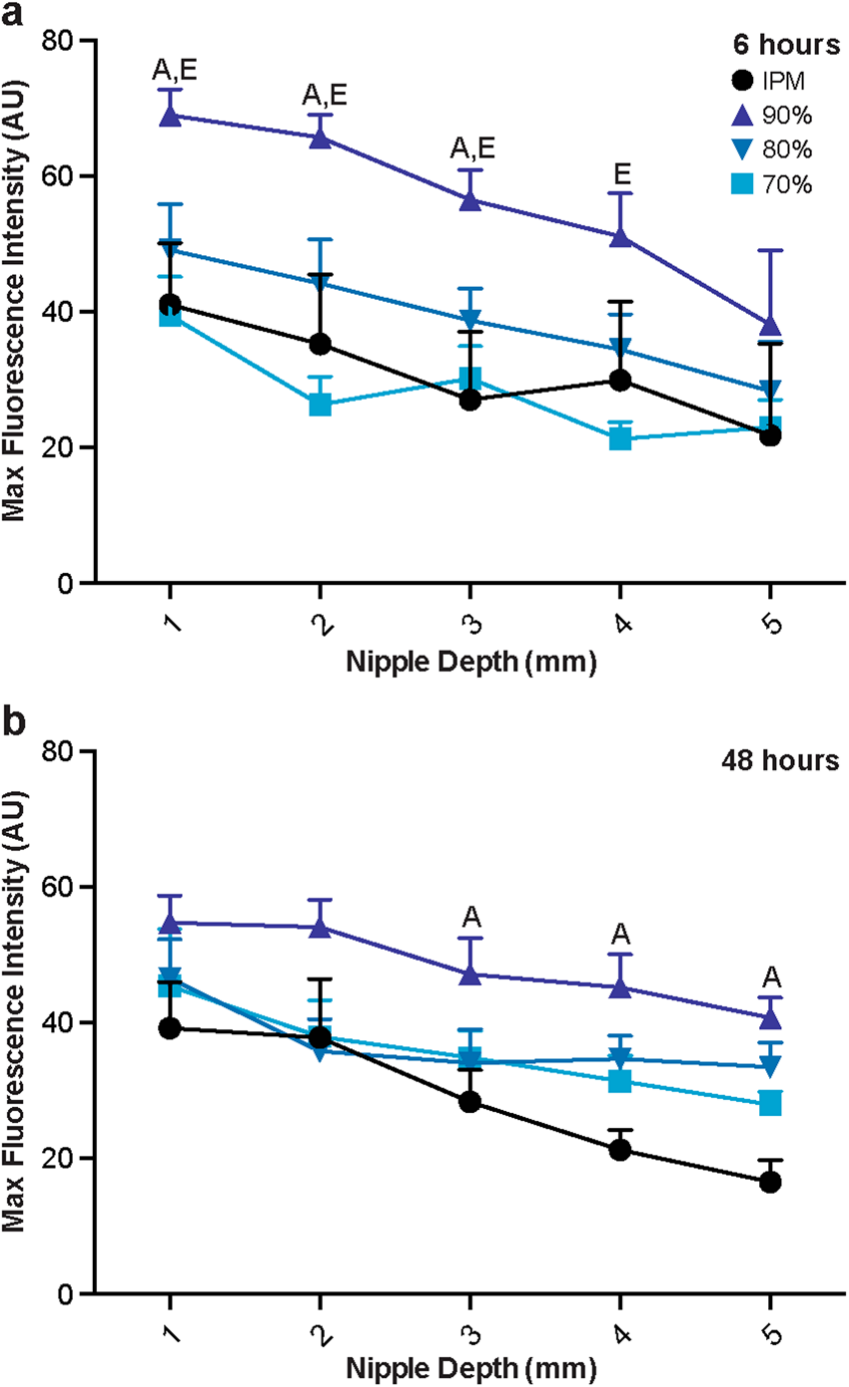
Peak fluorescence intensity of NR at the stratum corneum edge of porcine nipples treated with nanoemulsions or an IPM solution for 6 (**a**) or 48 (**b**) hours. Data is represented as mean + SEM (n = 4–6). Statistical analysis was performed with two-way ANOVA with Tukey’s multiple comparisons post-test. p ≤ 0.05 was considered significant and is denoted using the following indicators: A- IPM vs 90%, B- IPM vs 80%, C- IPM vs 70%, D- 90% vs 80%, E- 90% vs 70%, F- 80% vs 70%.

In comparison to the ductal penetration pathway, penetration via the stratum corneum yielded similar results in regard to differences among the treatments. Minimal differences were found in the stratum corneum fluorescence intensity profiles between the IPM solution and the 70% and 80% water nanoemulsion formulations following diffusion for 6 hours (Fig. 5a). Each of these treatments penetrated approximately 200 μm into the connective tissue at tissue depths 1–4 mm or 150 μm at a tissue depth of 5 mm. Furthermore, their peak fluorescence intensities at every nipple depth were not significantly different, each ranging from 40–50 at 1 mm in tissue depth and linearly decreasing as the tissue depth increased (Fig. 6a). However, the 90% water nanoemulsion penetrated slightly further in the top 3 mm, as evidenced by higher fluorescence intensity at a 200 μm penetration distance (Fig. 5a) and had significantly higher peak fluorescence intensities in the top 4 mm (Fig. 6a) compared to the other treatments following diffusion for 6 hours.

After diffusion for 48 hours, however, there were minimal differences among all of the treatments in stratum corneum fluorescence intensity profiles, similar to the ductal fluorescence intensity profiles at the same time point. In the top 2 mm, the penetration profiles for every treatment nearly overlap reaching penetration distances beyond 300 μm (Fig. 5b). In the bottom 3 mm, each treatment yielded a low level of fluorescence at 300 μm; however treatment with the 90% water nanoemulsion formulation resulted in higher fluorescence intensity within the connective tissue (>150 μm). Correspondingly, no significant differences in the peak fluorescence intensities were found between any of the treatments in the top 2 mm (Fig. 6b). From tissue depths between 3–5 mm, however, the peak fluorescence intensity of the 90% water nanoemulsion is significantly higher than the IPM solution.

Overall, the microscopic evaluation of the two transpapillary penetration routes indicates that the 90% water nanoemulsion was best able to penetrate the nipple via both the stratum corneum and mammary ducts, acting as an overall penetration enhancer. The 70% and 80% water nanoemulsions, however, showed moderate improvement in only the ductal penetration route, not the stratum corneum penetration route, suggesting these formulations more selectively target the mammary ducts.

### Stability of nanoemulsions upon topical nipple application

**Figure 7:**
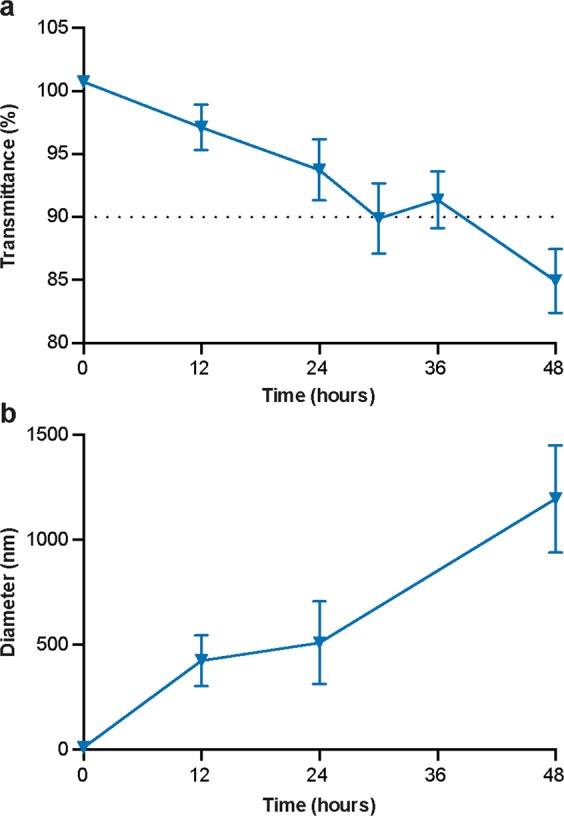
Stability of the 80% water nanoemulsion while in contact with porcine nipple tissue. The nanoemulsion formulation was applied topically on a Franz diffusion cell for up to 48 hours. Donor compartment samples were collected and measured for transparency (**a**) and droplet size. (**b**) Each data point represents mean ± SEM (n = 6).

Considering the shift from significant to minimal penetration differences among the nanoemulsion formulations and the control solution between 6 and 48 hours, the stability of nanoemulsions while in contact with porcine nipple tissue was assessed. The 80% water nanoemulsion formulation was applied topically on porcine nipples for up to 48 hours and the change in transmittance and droplet size was measured using techniques similar to those detailed by Patel *et al*.^34^. The transmittance linearly decreased while in contact with porcine nipple tissue, dropping below 90% after approximately 30 hours (Fig. 7a) at which point the solution was no longer considered a nanoemulsion^35^. Inversely, the droplet diameter and distribution rapidly increased following contact with the porcine tissue (Fig. 7b and Supplementary Fig. S6). Within 24 hours, the droplet diameter increased 49-fold from 10 to 509 nm. By 48 hours, the nanoemulsion droplet diameter was >1 μm, indicating transition to a particle larger than a nanoemulsion^16^. The impact of droplet destabilization on transpapillary penetration likely accounts for, in part, the disparity between the impact of nanoemulsion encapsulation at separate time points, as previous reports indicate transpapillary penetration decreases with an increase in molecular weight^11^ or drug vehicle diameter^11,28^. Therefore, as the nanoemulsion droplets increased in size, the penetration rate likely decreased, which hindered the overall formulation effects over time between 6 and 48 hours. While an increase in droplet size was observed, likely due to coalescence of the oil droplets over time, complete destabilization into two separate phases was not observed. Future studies will focus on enhancing formulation stability to optimize delivery efficiency.

## Conclusion

Nanoemulsions, consisting of IPM and T80, containing lipophilic fluorescent dye NR, successfully enhanced dye penetration into porcine nipple tissue *in vitro* in a formulation dependent manner. The nanoemulsion formulation with the highest water content (90%) markedly improved dye penetration via both the mammary ducts and the stratum corneum after diffusion for 6 hours. Meanwhile, the 70% and 80% water formulations moderately improved dye deposition via the ductal penetration route only, suggesting their targeting capabilities. The ability of nanoemulsion formulations to rapidly promote transport into the mammary ducts is an important practical consideration for drug delivery applications. Thus, future studies will broaden the nanoemulsion formulations considered with a focus on optimizing their stability and efficacy for transpapillary drug delivery.

## Materials and Methods

### Materials

Acetone, cytoseal60, ethanol, isoamyl alcohol, isopropyl myristate (IPM), Tween 80 (T80), nile red (NR) were purchased from Thermofisher. Abdominal porcine tissue was supplied from a local market and stored at −20 °C until use.

### Construction of pseudoternary phase diagram

The nanoemulsion region was determined by constructing a pseudoternary phase diagram using the water titration method^22^. IPM and T80 were used as the oil phase and surfactant, respectively. Isopropyl myristate and Tween 80 emulsion formulations have been used in transdermal delivery because of their ability to enhance skin permeation^20^, suggesting the potential advantage of their use for transpapillary delivery. First, IPM and T80 were mixed at varying w:w ratios, e.g. 1:9, 2:8, 3:7 etc. Then, distilled water was added incrementally to the oil and surfactant mixture. The samples were vortexed for one minute and kept at room temperature overnight before the next water titration. At each titration, the emulsion mixtures were visually inspected and categorized as a nanoemulsion when they appeared transparent and fluid.

### Preparation of NR nanoemulsions

First, varying concentrations of NR were solubilized in IPM, in some instances aided by vortexing and heating. Appropriate amounts of IPM containing NR and T80 were weighed into a glass vial and vortexed for one minute. Then, water was added drop-wise to the oil-surfactant mixture and again vortexed. The final nanoemulsion formulations (see Table 1) each had a final NR concentration of 0.04 mM.

### Characterization of nanoemulsions

The droplet size, polydispersity index (PDI), and zeta potential were measured by dynamic light scattering using a Nanobrook 90Plus PALS at room temperature (25 °C). The refractive index of water (1.331) was used for measurements considering the nanoemulsion formulations contained 70–90% water. The reported data is an average of the particle intensity values from quintuplicate measurements of three different preparations. CryoTEM was used to confirm the droplet size. A 4 μL sample was applied to a carbon mesh 200 copper grid, blotted and then flash frozen in liquid ethane. Images were acquired using a FEI Tecnai G2 F30 transmission microscope.

### *In vitro* nipple permeation study

Porcine nipple tissue was supplied from a local market and stored at −20 °C. Prior to use, tissue was thawed and underlying dermal fat was removed. The nipple was then sandwiched between the donor and receptor compartments of a Franz diffusion cell for 6 or 48 hours. The donor compartment was filled with 400 μL of the nanoemulsion formulation or control solution (IPM) incorporating NR at 0.04 mM. IPM was chosen as the control solvent due to its relevance as the nanoemulsion’s oil phase as well as NR’s limited solubility in an aqueous solution^36^. The receptor compartment was filled with 5 mL of PBS or IPM, stirred using a magnetic stir bar, and maintained at 37 °C. Different solutions were used in the receptor compartment based on NR’s solubility and potential to diffuse across the entire tissue and reach the receptor chamber. Because numerous studies indicate that the lag time across the nipple is minimally 11 hours^9–11^, the receptor compartment was filled with PBS for the 6-hour treatments. For the 48-hour experiments, during which it was more likely that the NR would diffuse across the entire length of the nipple and into the receptor chamber, the receptor chamber was filled with IPM. After specified treatment times, the nipple was removed from the Franz diffusion cell and the surface was rinsed with 3 mL of ethanol before storage at −20 °C until further processing.

### Quantification of dye retention in nipple

NR was extracted from 1-mm tissue sections as previously reported^9,28^. Briefly, frozen nipples were sectioned on a cryostat from tip to base in 100-μm increments. Every 1 mm, tissue was collected in a storage tube and the tissue weight was determined. Dye was then extracted by adding 350 μL of 6:1 isoamyl alcohol:acetone (v:v) to each tube and incubated at room temperature for at least 30 min. The dye concentration was determined by interpolation from a standard curve following fluorescence measurement on a Synergy H1 fluorimeter at 550/630 nm excitation/emission.

### Fluorescence imaging and image analysis

During nipple sectioning, a 20-μm section was taken every 1 mm, mounted on a slide, and incubated at 37 °C for at least 6 hours before sealing with Cytoseal60. Fluorescence micrographs were obtained using a Zeiss Axioplan 2 with a Cy3 filter set at 10x magnification. All images were taken at the same exposure and with similar image renormalization to minimize image data variation. Numerous images were taken across the entire tissue section and subsequently stitched together to generate a montage of the entire nipple cross-section (Fig. 2A). The plot profile tool in ImageJ^37^ was then used to calculate the fluorescence intensity as a function of distance (Fig. 2B,C). Five to eight profiles were drawn normal to the ductal edge (ductal penetration) or stratum corneum edge (stratum corneum penetration) per tissue section and averaged (n = 6 tissue sections per treatment formulation/time combination). This analysis was performed at consecutive increments up to 5.0 mm in nipple depth. A schematic outlining transpapillary penetration in the x-y-z plane is illustrated previously in Kurtz *et al*.^28^.

### Stability of nanoemulsion on nipple tissue

Nanoemulsion stability during topical application on the nipple was studied as a function of transmittance and droplet size. Nipples were arranged in a Franz diffusion cell for up to 48 hours as described in the *in vitro* nipple permeation study. For transmittance measurements, 10 μL of the donor solution was removed at pre-specified time points and the absorbance (A) at 650 nm was measured in triplicate using a UV spectrophotometer (NanoDrop2000). The percent transmittance (T) was then calculated using the equation T = 10^(2-A)^. To determine the droplet size, samples of the donor solution were collected, diluted 1:10–1:25 (v:v) with distilled water, and immediately analyzed using dynamic light scattering (NanoBrook 90Plus PALS).

### Statistical analysis

GraphPad Prism software version 7 (San Diego, CA) was used for all statistical analysis. A one-way ANOVA or two-way ANOVA with Tukey’s multiple comparison post-test was used for analysis of total retained dye or analysis of treatment by depth, respectively. All results were deemed significant when p ≤ 0.05.

## Supplementary information

Supplementary Information

## Data Availability

The datasets generated and/or analyzed during the current study are available from the corresponding author on request.
