# Supplementary material for: Nanoemulsions Enhance in vitro Transpapillary Diffusion of Model Fluorescent Dye Nile Red
Source: Sci Rep. 2019 Aug 14;9:11810. doi: 10.1038/s41598-019-48144-x (PMC6694173; doi:10.1038/s41598-019-48144-x)
Supplement: Supplementary file 1 — Supplementary Information [file 41598_2019_48144_MOESM1_ESM.docx]

**SUPPLEMENTARY INFORMATION**

**Nanoemulsions Enhance *in vitro* Transpapillary Diffusion of Model Fluorescent Dye Nile Red**

Samantha L. Kurtz^a,b^, Louise B. Lawson^a^*

^a^Department of Microbiology and Immunology, Tulane University School of Medicine, New Orleans, LA 70112 USA

^b^Bioinnovation Ph.D. Program, Tulane University School of Science and Engineering, New Orleans, LA 70118 USA

*Corresponding author: Mailing address: Department of Microbiology and Immunology, Tulane University School of Medicine, 1430 Tulane Ave (8638), New Orleans, LA 70112, USA. Tel.: (504)988-2204; Fax: (504)988-5144

*E-mail Address*: [lbraud@tulane.edu](mailto:lbraud@tulane.edu) (L.B. Lawson)


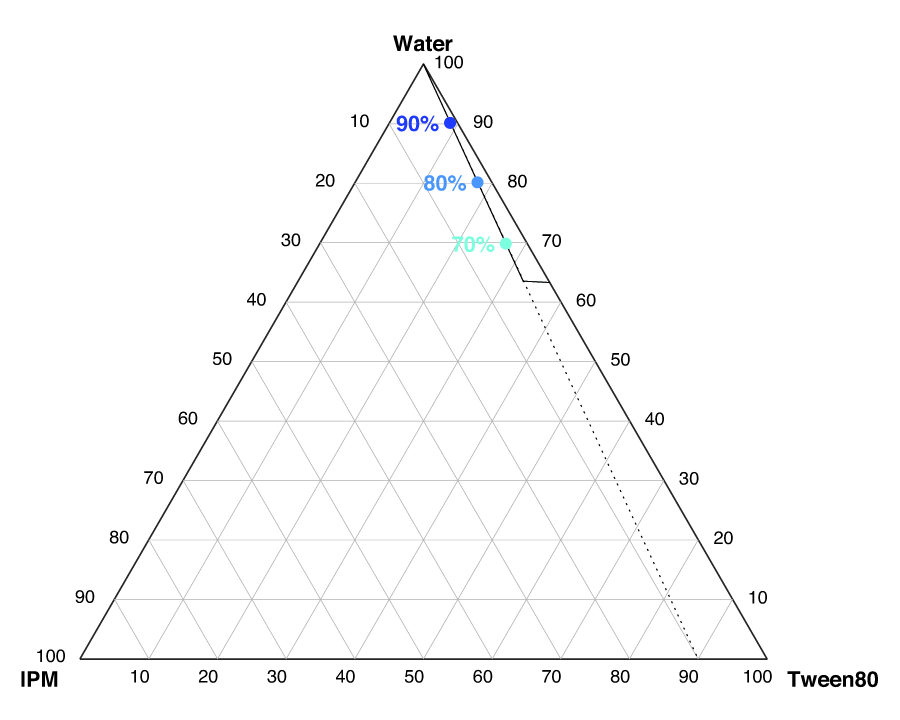


**Fig. S1** A phase diagram of the IPM-T80-water mixture, with the boxed area indicating the nanoemulsion region chosen for characterization. The labeled points denote the nanoemulsion formulations that were used in *in vitro* nipple permeation studies.


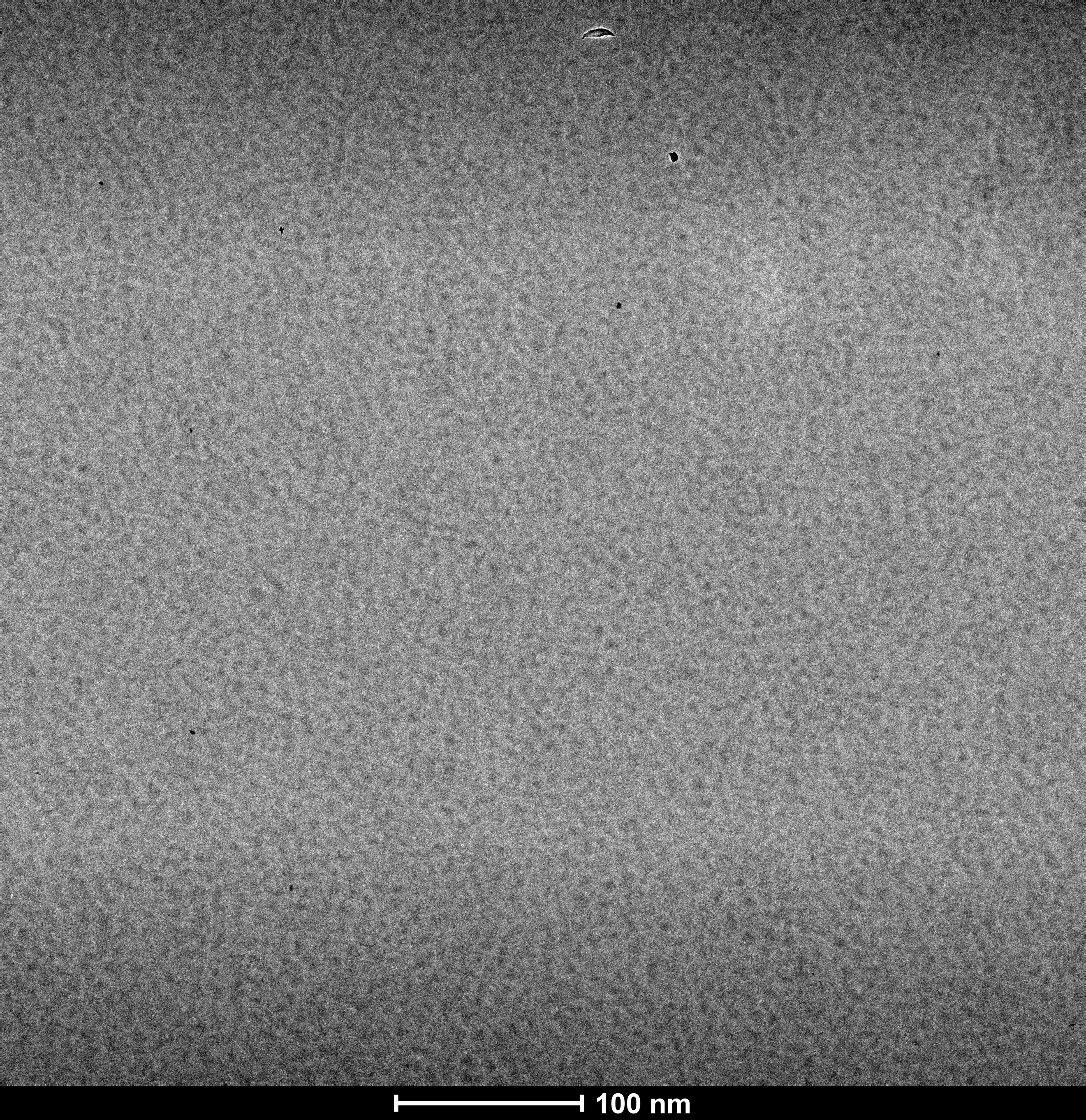


**Fig. S2** A cryoTEM image of the 80% water nanoemulsion formulation.


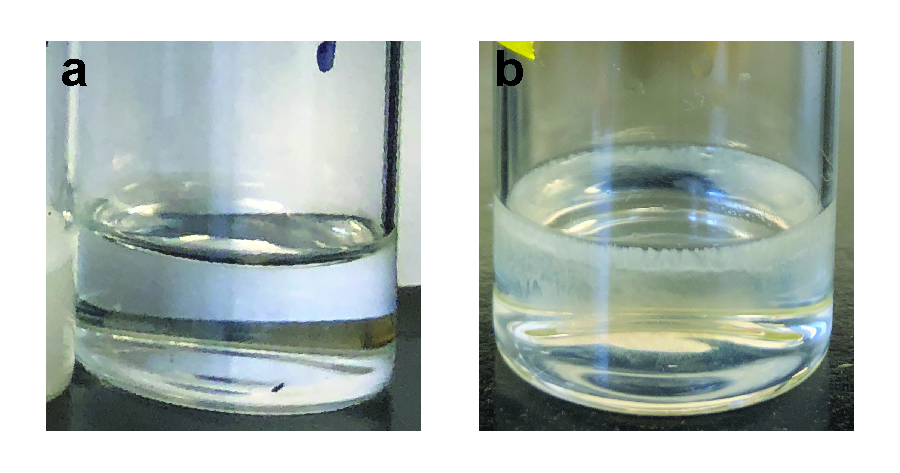


**Fig. S3** Representative images of the 80% water nanoemulsion formulation following storage for 24 hours (a) or 11 months (b) at room temperature. After 24 hours, the preparation is isotropic and fluid. However after 11 months, the preparation exhibits phase separation.


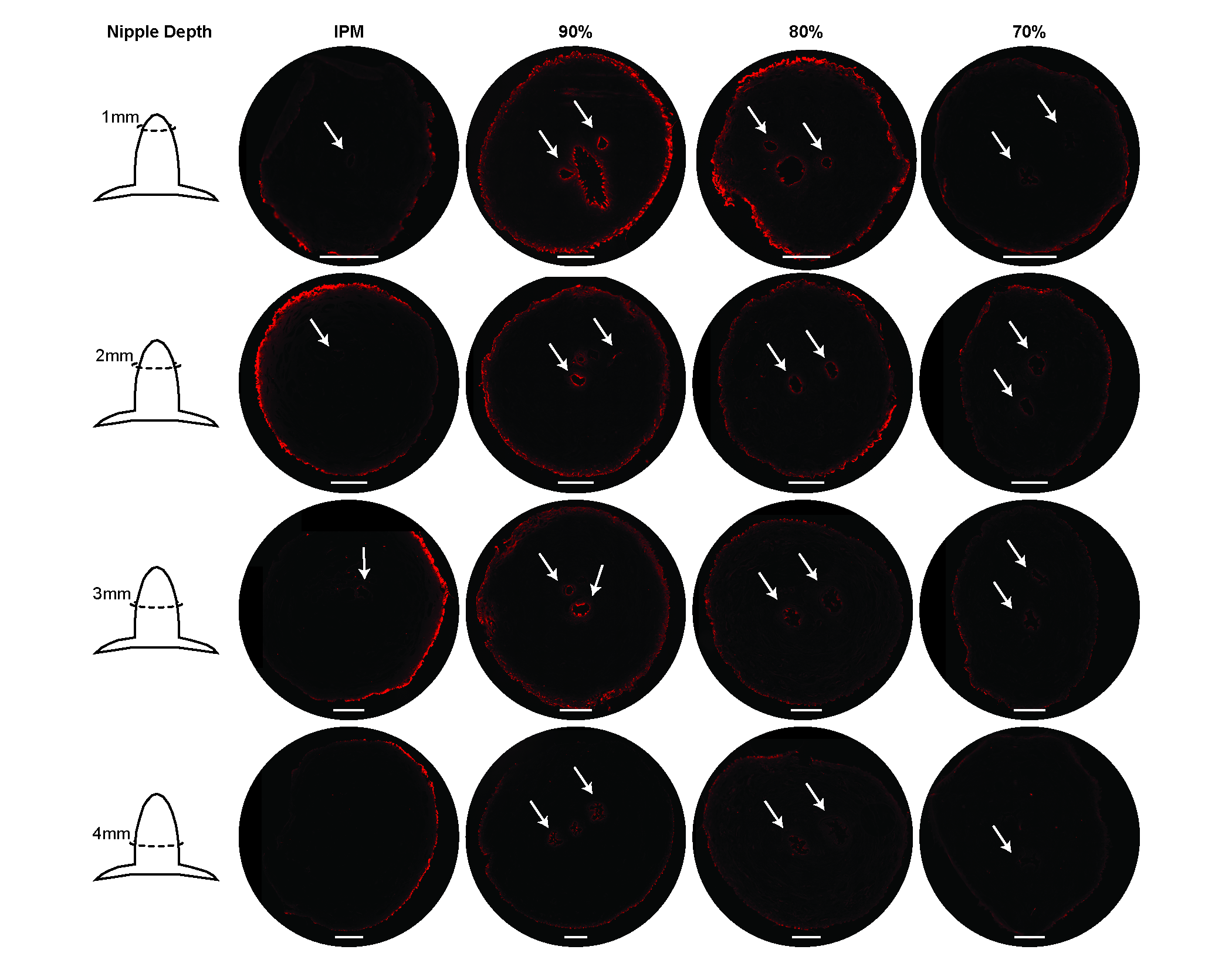


**Fig. S4** Representative fluorescence micrographs of nipple cross-sections following diffusion of nanoemulsion formulations or the control IPM solution for 6 hours. White scale bar represents 1 mm. White arrows indicate mammary ducts.


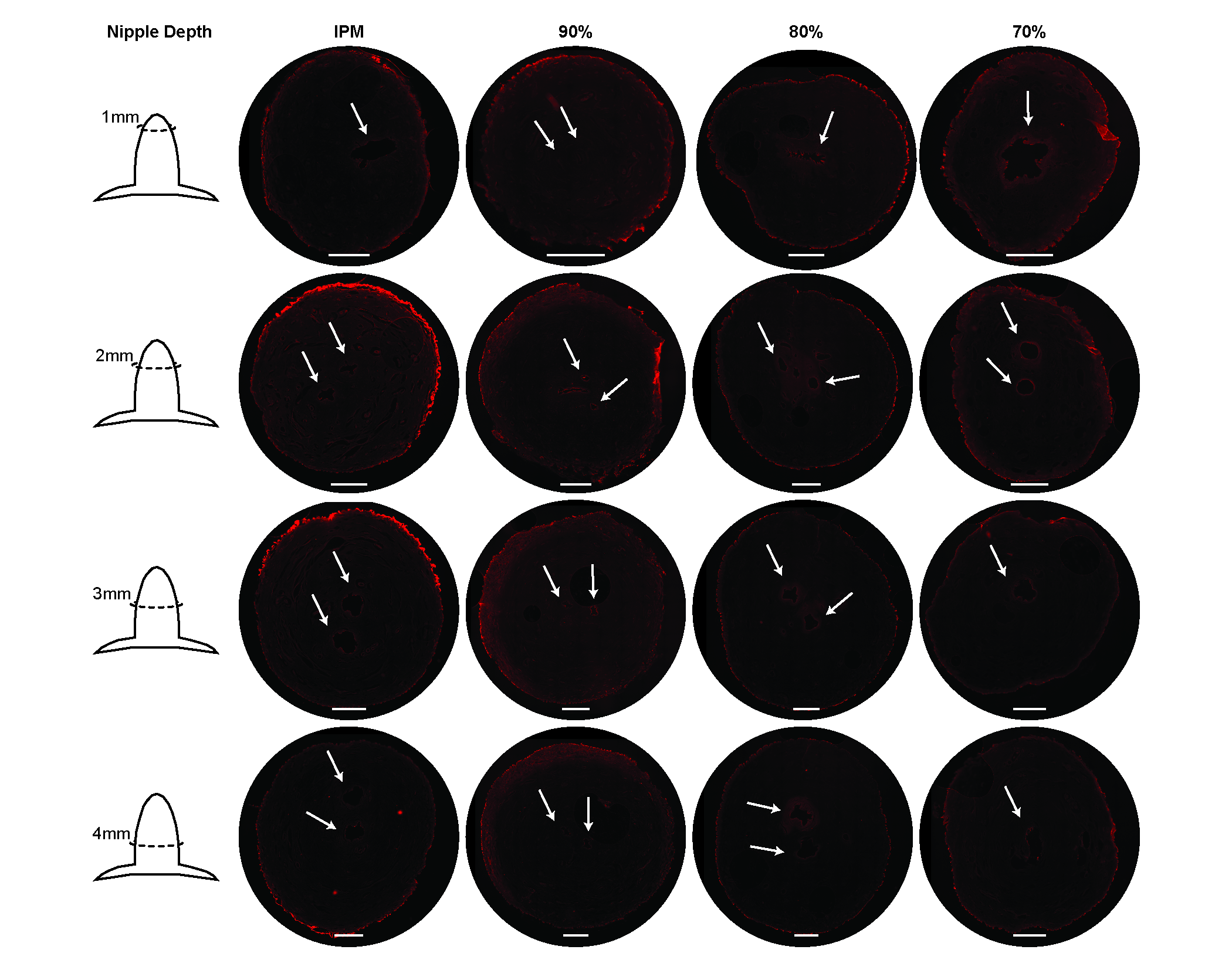


**Fig. S5** Representative fluorescence micrographs of nipple cross-sections following diffusion of nanoemulsion formulations or the control IPM solution for 48 hours. White scale bar represents 1 mm. White arrows indicate mammary ducts.


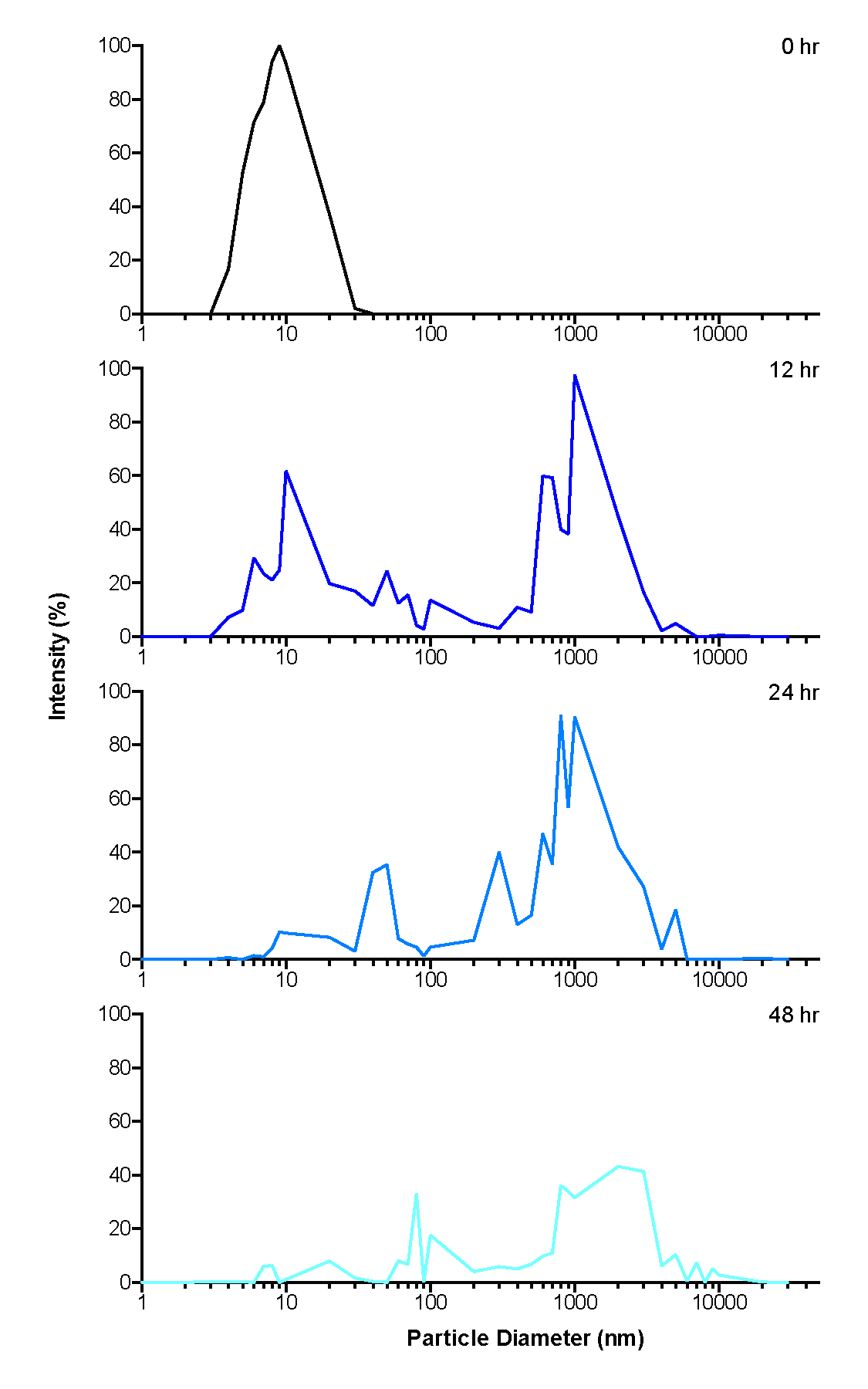


**Fig. S6** Size distribution of the 80% water formulation following topical nipple application for 0-48 hours as measured by DLS. Each line is an average of 3-6 permeations measured in quintuplicate.
